# Evidence that the presynaptic vesicle protein CSPalpha is a key player in synaptic degeneration and protection in Alzheimer’s disease

**DOI:** 10.1186/s13041-015-0096-z

**Published:** 2015-01-29

**Authors:** Sachin S Tiwari, Marie d’Orange, Claire Troakes, Badrun N Shurovi, Olivia Engmann, Wendy Noble, Tibor Hortobágyi, Karl P Giese

**Affiliations:** Centre for Cellular Basis of Behaviour, Department of Neuroscience, King’s College London, 125 Coldharbour Lane, London, SE5 9NU UK; Department of Clinical Neuroscience, Institute of Psychiatry, King’s College London, De Crespigny Park, London, SE5 8AF UK; Department of Neuroscience, King’s College London, DeCrespigny Park, London, SE5 8AF UK; Department of Neuropathology, Institute of Pathology, University of Debrecen, 4032 Debrecen, Hungary; Centre for the Cellular Basis of Behaviour, James Black Centre, King’s College London, Institute of Psychiatry, 125 Coldharbour Lane, London, SE5 9NU UK

**Keywords:** Alzheimer’s disease, Cerebellum, Cysteine string protein, Hippocampus, Synapses, Neuroprotection

## Abstract

**Background:**

In Alzheimer’s disease synapse loss precedes neuronal loss and correlates best with impaired memory formation. However, the mechanisms underlying synaptic degeneration in Alzheimer’s disease are not well known. Further, it is unclear why synapses in AD cerebellum are protected from degeneration. Our recent work on the cyclin-dependent kinase 5 activator p25 suggested that expression of the multifunctional presynaptic molecule cysteine string protein alpha (CSPalpha) may be affected in Alzheimer’s disease.

**Results:**

Using western blots and immunohistochemistry, we found that CSPalpha expression is reduced in hippocampus and superior temporal gyrus in Alzheimer’s disease. Reduced CSPalpha expression occurred before synaptophysin levels drop, suggesting that it contributes to the initial stages of synaptic degeneration. Surprisingly, we also found that CSPalpha expression is upregulated in cerebellum in Alzheimer’s disease. This CSPalpha upregulation reached the same level as in young, healthy cerebellum. We tested the idea whether CSPalpha upregulation might be neuroprotective, using htau mice, a model of tauopathy that expresses the entire wild-type human tau gene in the absence of mouse tau. In htau mice CSPalpha expression was found to be elevated at times when neuronal loss did not occur.

**Conclusion:**

Our findings provide evidence that the presynaptic vesicle protein CSPalpha is a key player in synaptic degeneration and protection in Alzheimer’s disease. In the forebrain CSPalpha expression is reduced early in the disease and this may contribute to the initial stages of synaptic degeneration. In the cerebellum CSPalpha expression is upregulated to young, healthy levels and this may protect cerebellar synapses and neurons to survive. Accordingly, CSPalpha upregulation also occurs in a mouse model of tauopathy only at time when neuronal loss does not take place.

**Electronic supplementary material:**

The online version of this article (doi:10.1186/s13041-015-0096-z) contains supplementary material, which is available to authorized users.

## Introduction

Alzheimer’s disease is a devastating neurodegenerative condition and the most prominent cause of dementia. The neuropathological features of Alzheimer’s disease are substantial neuronal death in the forebrain, but almost no neurodegeneration in the cerebellum [1,2]. In the forebrain extracellular amyloid plaques and intracellular neurofibrillary tangles are characteristic of Alzheimer’s disease. Further, synaptic loss precedes neuronal loss and the former correlates best with early deficits in memory formation [3,4]. Our recent research provided a novel window into the mechanisms underlying synaptic degeneration in Alzheimer’s disease [5]. We found that the truncated cyclin-dependent kinase 5 activator p25 is reduced in Alzheimer’s disease [6]. Normally, p25 generation is linked to the synthesis of particular synaptic proteins, synaptogenesis and memory formation [6]. Thus, impaired p25 generation may cause early synaptic dysfunction in Alzheimer’s disease. Furthermore, we demonstrated that p25 generation regulates expression of the synaptic chaperone protein cysteine string protein (CSP) alpha [6]. CSPalpha is a synaptic vesicle protein that belongs to a conserved gene family [7,8] that includes CSPalpha, CSPbeta and CSPgamma of which only CSPalpha is expressed in the brain [9,10]. CSPalpha function is essential for synaptic survival as indicated in mouse knockout studies [10]. Furthermore, loss-of-function CSPalpha mutations are responsible for autosomal dominant Kufs disease, an adult-onset neurodegenerative disorder with associated dementia [11,12]. CSPalpha is proposed to serve various functions at the presynapse, including: 1) Formation of a trimeric complex with SGT and Hsc 70, resulting in a CSP/SGT/Hsc70 chaperone complex that is localised at synaptic vesicles [13] and interacts with SNARE proteins leading to calcium-triggered synaptic vesicle exocytosis [14,15]. 2) Modulation of presynaptic calcium levels by regulating the activity of presynaptic calcium channels [8,16]. 3) Regulation of endocytosis by facilitating dynamin 1 polymerization [17]. 4) Regulation of the density of calcium-dependent K^+^ (BK) channel at the presynaptic terminal, controlling the excitability there [18,19].

Our finding that CSPalpha is a p25-regulated protein [6] suggested that CSPalpha expression may be impaired in Alzheimer’s disease. Here we tested this hypothesis by examination of post-mortem human tissues. As expected, we found that CSPalpha expression is reduced in forebrain of early and late Alzheimer’s disease. Interestingly, CSPalpha expression was reduced before synaptophysin levels drop, suggesting that it contributes to the initial stages of synaptic degeneration. Surprisingly, we discovered an upregulation of CSPalpha expression in Alzheimer’s disease cerebellum, a brain area that is protected from synaptic degeneration. Further post-mortem investigations and work with a mouse model of tauopathy established a novel correlation between CSPalpha upregulation and neuroprotection.

## Results

### Specificity of anti-CSPalpha antibody

**Figure 1:**
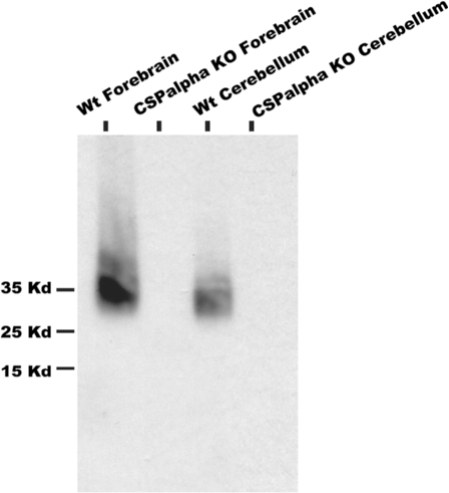
**Specificity of the anti-CSPalpha antibody used in this study.** The anti-CSPalpha antibody did not react with any protein in forebrain and cerebellum from CSPalpha knockout (KO) mice. In wild-type mice (WT) the antibody recognized a smear of bands at an approximate molecular weight of 35 kiloDalton, indicating complex postranslational modification of CSPalpha.

To study CSPalpha protein expression we performed western blots and immunohistochemistry with an anti-CSPalpha antibody, which does not react with other protein in CSPalpha knockout mice [10] (Figure 1).

### CSPalpha expression is reduced in post-mortem Alzheimer’s disease hippocampus

**Figure 2:**
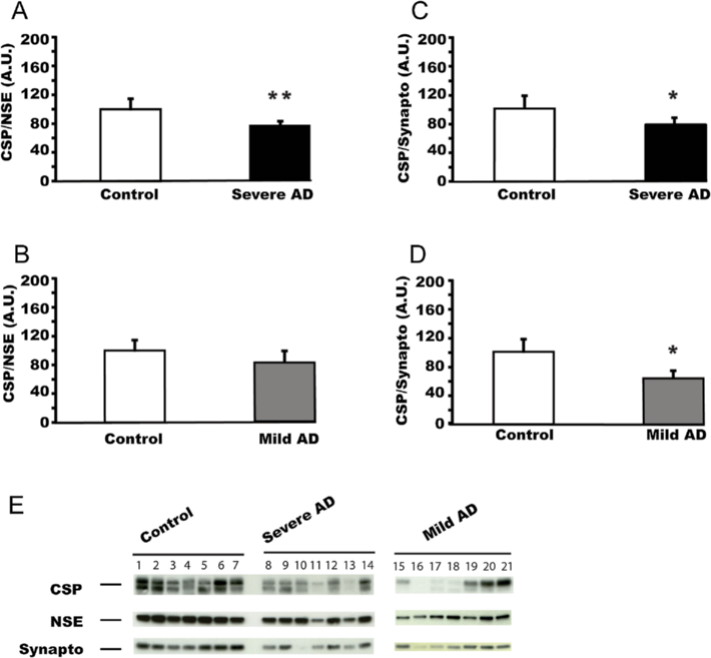
**CSPalpha protein expression is reduced in Alzheimer’s disease hippocampus. (A)** CSPalpha expression in post-mortem hippocampus from patients with severe Alzheimer’s disease (Braak stages 5 and 6; n = 12; average age at death = 75.2 ± 2.0 years) and control subjects (n = 12; average age at death = 76.5 ± 2.9 years). CSPalpha expression was normalized against the neuron-specific house keeping marker protein NSE. **(B)** CSPalpha expression in post-mortem hippocampus from patients with mild Alzheimer’s disease (Braak stages 1 and 2; n = 12; average age at death = 80.3 ± 3.2 years) and control subjects (n = 12). CSPalpha expression was normalized against NSE. **(C)** The same samples as in panel **(A)** were used but CSPalpha expression was normalized against the synaptic marker protein synaptophysin (severe Alzheimer’s disease, n = 11; control, n = 11). **(D)** The same samples as in panel **(B)** were used but CSPalpha expression was normalized against synaptophysin (mild Alzheimer’s disease, n = 12; control, n = 11). Panel **(E)** shows the representative western blots for 7 controls, 7 patients with severe AD and 7 patients with mild AD. Note that the anti-CSPalpha antibody recognizes two bands at an approximate molecular weight range of 35 kiloDalton, which are likely to represent distinct post-translational modifications of CSPalpha. Means ± s.e.m. are shown. *, p < 0.05; **, p < 0.01.

The hippocampus is one of the earliest and one of the most severely affected brain regions in Alzheimer’s disease [20]. We studied whether CSPalpha protein expression is affected in this brain region, comparing post-mortem samples from severe Alzheimer’s disease patients (Braak stages 5 and 6) and age-matched control subjects by western blot analysis. CSPalpha expression was normalized to either the neuronal marker NSE or the synaptic marker synaptophysin (Figure 2A, C). In both cases CSPalpha levels were significantly decreased in the hippocampus of severe Alzheimer’s disease patients (referring to NSE expression: F(2,21) = 21.3; p < 0.01; referring to synaptophysin expression: F(2,19) = 14.6, p < 0.05). This result suggests that in severe Alzheimer’s disease CSPalpha expression is not simply reduced as a result of neuronal or synaptic loss, and that reduced CSPalpha expression may precede synaptic loss during the progression of Alzheimer’s disease.

Interestingly, we did not find that synaptopysin levels are reduced in Alzheimer’s disease when normalized to NSE expression (Additional file 1: Figure S1). In contrast synaptophysin levels are reduced when absolute expression levels are considered (e.g., [21-23]). Therefore, neuronal loss in Alzheimer’s disease appears to mainly contribute to reduced expression of synaptophysin, which can be corrected for when expression is normalized to NSE.

We also analysed CSPalpha expression in mild Alzheimer’s disease (Braak stages 1 and 2) (Figure 2B, D). Analysis of western blot results revealed a significant decrease in CSPalpha levels in the mild Alzheimer’s disease hippocampus when normalized with synaptophysin (F(2,20 = 4.26; P < 0.05), but showed no difference when normalized with NSE (F(2,21 = 0.366, p = 0.427). These results indicate that decreases in CSPalpha expression in the hippocampus is an early event in Alzheimer’s disease.

### CSPalpha expression is reduced in Alzheimer’s disease superior temporal gyrus

**Figure 3:**
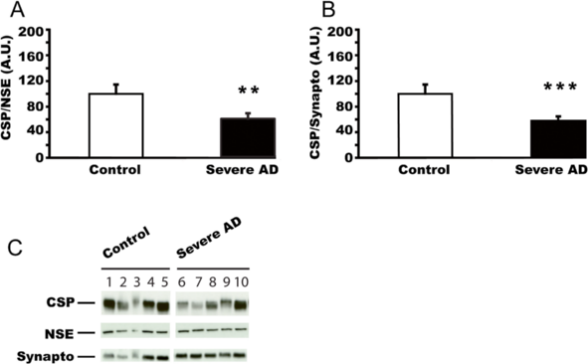
**CSPalpha protein expression is reduced in Alzheimer’s disease superior temporal gyrus. (A)** CSPalpha expression in post-mortem STG from patients with severe Alzheimer’s disease (n = 13; average age at death = 73.2 ± 3.4 years) and control subjects (n = 12; average age at death = 76.9 ± 2.1 years). CSPalpha expression was normalized against NSE. **(B)** The same samples as in panel **(A)** were used but CSPalpha expression was normalized against synaptophysin (severe Alzheimer’s disease, n = 13; control, n = 12). Panel **(C)** shows the representative western blots for 5 controls and 5 patients with severe AD. Means ± s.e.m. are shown. **, p < 0.01; ***, p < 0.001.

We next studied CSPalpha expression in the superior temporal gyrus (STG), which is affected later and less severely than the hippocampus in Alzheimer’s disease. In a western blot analysis we compared CSPalpha expression in severe Alzheimer’s disease and control STG. The level of CSPalpha expression was again normalised to NSE or synaptophysin (Figure 3). In both cases, there was a significant reduction in CSPalpha expression (referring to NSE expression: F(2,22) = 14.8; p < 0.01; referring to synaptophysin expression: F(2,22) = 7.48, p < 0.001), similar to that found in the hippocampus (Figure 2A, C). These results show that changes in CSP levels are not limited to the hippocampus in Alzheimer’s disease brain, but are also found in other degenerating forebrain regions.

### CSPalpha expression is increased in Alzheimer’s disease cerebellum

**Figure 4:**
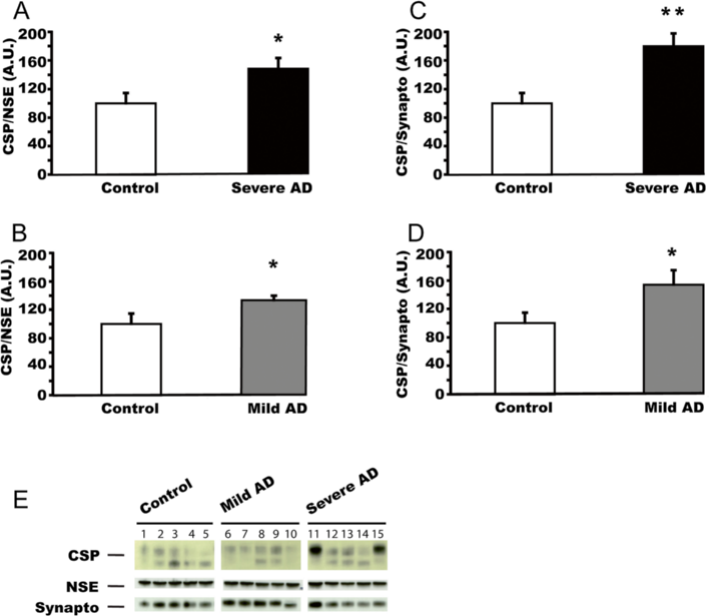
**CSPalpha expression is increased in Alzheimer’s disease cerebellum. (A)** CSPalpha expression in post-mortem cerebellum from patients with severe Alzheimer’s disease (n = 10; average age at death = 74.7 ± 4.0 years) and control subjects (n = 10; average age at death = 76.3 ± 4.2 years). CSPalpha expression was normalized against NSE. **(B)** CSPalpha expression in post-mortem cerebellum from patients with mild Alzheimer’s disease (n = 10; average age at death = 81.3 ± 4.1 years) and control subjects (n = 10). CSPalpha expression was normalized against NSE. **(C)** The same samples as in panel **(A)** were used but CSPalpha expression was normalized against synaptophysin. **(D)** The same samples as in panel **(B)** were used but CSPalpha expression was normalized against synaptophysin. Panel **(E)** shows the representative western blots for 5 controls, 5 patients with mild AD and 5 patients with severe AD. Means ± s.e.m. are shown. *, p < 0.05; **, p < 0.01.

The cerebellum is the least affected brain structure in Alzheimer’s disease [1,2]. There is no synapse and neuronal loss in this brain region in this disease. We investigated the levels of CSPalpha expression in cerebellum from severe Alzheimer’s disease and controls, using western blot analysis. As in the case of hippocampus, CSPalpha amounts were normalized against NSE and synaptophysin (Figure 4A, C). In both cases CSPalpha levels were significantly increased by about 50% (referring to NSE expression: F(2,17) = 2.76; p < 0.05; referring to synaptophysin expression: F(2,17) = 5.28, p < 0.01). We also analysed CSPalpha expression in mild Alzheimer’s diseaseand control cerebellum (Figure 4B, D). We found that the level of CSPalpha expression was significantly increased in mild Alzheimer’s disease cerebellum when normalized with both NSE and synaptophysin (referring to NSE expression: F(2,17) = 7.25; p < 0.05; referring to synaptophysin expression: F(2,17) = 4.18, p < 0.05). Therefore, we have observed reduced CSPalpha amounts in degenerating regions of early and severe Alzheimer’s disease brain, and increased levels of CSPalpha in areas of Alzheimer’s disease brain that are relatively spared from degeneration. These findings suggest that there may be a mechanistic link between CSPalpha expression levels and neuroprotection in Alzheimer’s disease.

### Immunohistochemical analysis confirms CSPalpha downregulation in forebrain and upregulation in cerebellum in Alzheimer’s disease

**Figure 5:**
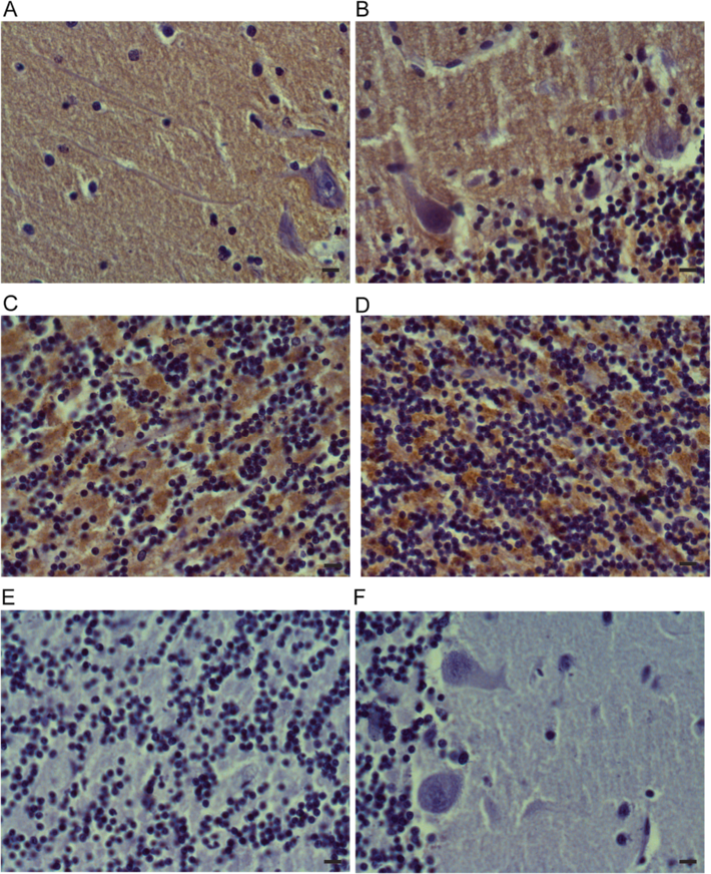
**Increased CSPalpha immunostaining in Alzheimer’s disease cerebellum.** Fixed cerebellar cortex sections from a patient with severe Alzheimer’s disease **(B, D)** and a control subject **(A, C)** were probed with anti-CSPalpha antibodies for analysis of CSPalpha expression. CSPalpha expression in the cerebellar cortex of the Alzheimer’s disease patient appeared higher than in the control subject. In **A** and **B** cerebellar cortex with the Purkinje cell layer and granule cells are visible in the lower right corner. There is increased immunoreactivity in the neuropil in Alzheimer’s disease as compared to an age-matched control case. In **C** and **D** the granule cells of the cerebellum show increased cytoplasmic and neuropil labeling in Alzheimer’s disease as compared to an age-matched control. Negative control images are shown in **E** and **F** (immunohistochemistry with omission of the primary antibody) and confirm the specificity of labeling in Figure 5 and Additional file 1: Figure S1. Haematoxylin counterstaining is also shown. Original magnification: 400×. Scale bars represent 20 μm.

To confirm the changes in CSPalpha protein amounts determined by western blotting, we carried out a qualitative immunohistochemical analysis with post-mortem cerebellum, hippocampus and STG from a severe Alzheimer’s disease patients and a control subject (Figure 5; Additional file 2: Figure S4). A neuropathologist blinded to the disease state of the tissue performed a qualitative comparison of CSPalpha expession. This comparison confirmed an increase in CSPalpha levels in cerebellar regions (Figure 5) and a decrease in hippocampus and STG in severe Alzheimer’s disease compared to control (Additional file 2: Figure S4). To validate the synaptic specificity of the anti-CSPalpha antibodies, the immunohistochemistry was compared with synaptophysin expression patterns (Additional file 3: Figure S2). Staining of cerebellar dentate nucleus from a control subject showed similar results with both antibodies, confirming that the CSPalpha immunostaining was typical for synaptic staining.

### CSPalpha expression is not changed in frontotemporal lobar degeneration (FTLD) cerebellum

**Figure 6:**
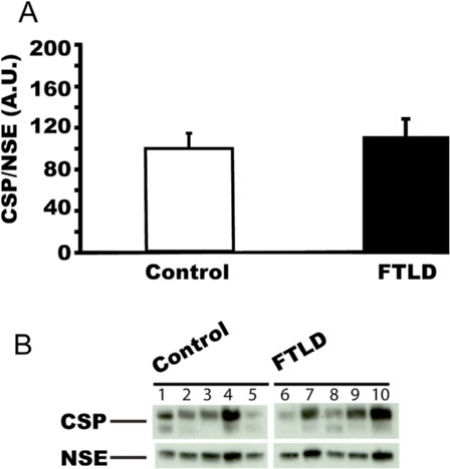
**CSPalpha expression in cerebellum is not changed in FTLD.** CSPalpha expression in post-mortem cerebellum from patients with FTLD (n = 5; average age at death = 73.0 ± 3.0 years) and control subjects (n = 5; average age at death = 70.2 ± 7.2 years).CSPalpha expression was normalized against NSE **(A)**. Panel **(B)** shows the representative western blots for 5 controls and 5 patients with FTLD. Means ± s.e.m. are shown.

Frontotemporal lobar degeneration (FTLD) is a prominent form of dementia that is characterized by neurodegeneration of the frontotemporal region [24]. In addition to frontal lobe degeneration, the cerebellum is also reported to be affected in FTLD cases [25]. This disparity provided an opportunity to test the specificity of our observation that CSPalpha is upregulated in Alzheimer’s disease cerebellum. We performed a western blot analysis to study whether altered CSPalpha expression is linked to cerebellar neuropathology in FTLD (Figure 6). CSPalpha expression levels did not differ between FTLD and control cerebellum (t = − 0.373, p = 0.72). Hence, increased CSPalpha expression in the cerebellum is not a common feature of neurodegenerative disease, but appears to be specific for Alzheimer’s disease.

### Normal ageing reduces CSPalpha expression in human cerebellum

**Figure 7:**
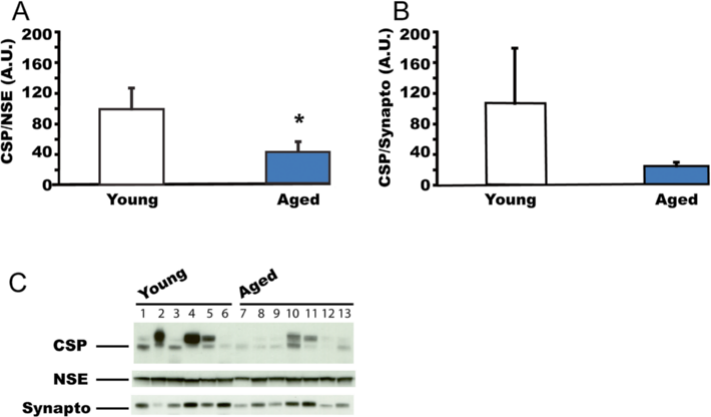
**Normal ageing reduces CSPalpha expression in healthy human cerebellum. (A)** CSPalpha expression in post-mortem cerebellum from healthy young (n = 6; average age at death = 21.3 ± 1.6 years) and aged (n = 7; average age at death = 96.1 ± 1.4 years) subjects. CSPalpha expression was normalized against NSE. **(B)** The same samples as in panel **(A)** were used but CSPalpha expression was normalized against synaptophysin. Panel **(C)** shows the representative western blot for 6 young subjects and 7 aged subjects. Means ± s.e.m. are shown. *, p < 0.05.

The difference in CSPalpha expression in the hippocampus and cerebellum of Alzheimer’s disease brain suggests that CSPalpha expression may be differentially regulated under physiological and/or pathological conditions. Therefore, we studied whether normal ageing also regulates CSPalpha expression in cerebellum. Using western blots, we analysed CSPalpha protein expression in cerebellum from healthy subjects belonging to two age groups, 15–30 years (21.3 ± 1.6 years) and 90–105 years (96.1 ± 1.4 years) (Figure 7). CSPalpha expression was reduced by approximately 50% in the aged cerebellum when expression was normalized to NSE expression (t = 2.443; p < 0.05). Similarly, when normalized to synaptophysin levels the average CSPalpha expression in the aged cerebellum appeared lower than in young cerebellum, although this did not reach significance (t = 1.351; p = 0.23), most likely due to a large variability of CSPalpha amounts in young cerebellum. Taken together, our results suggest that CSPalpha amounts are subject to age-dependent decreases in healthy cerebellum. Interestingly, the CSPalpha expression level in Alzheimer’s disease cerebellum is similar to the expression level found in young healthy cerebellum.

### Overexpression of human tau leads to an age-dependent decline in hippocampal-cortical CSPalpha levels

**Figure 8:**
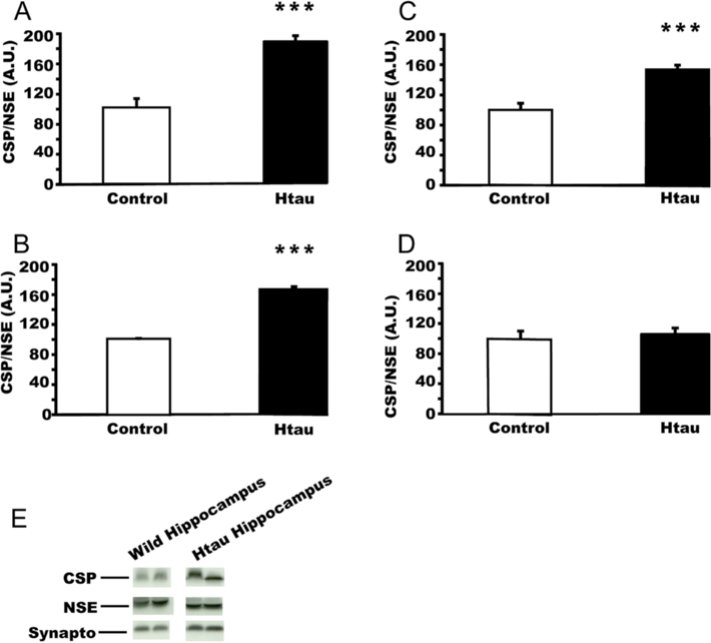
**Mice expressing human tau have an age-dependent CSPalpha upregulation that correlates with neuroprotection. (A)** CSPalpha expression in hippocampus of young (3–4 months) wild type (n = 6) and htau mice (n = 6). **(B)** CSPalpha expression in cortex of young (3–4 months) wild type (n = 6) and htau mice (n = 6). **(C)** CSPalpha expression in cerebellum of the same mouse sample as in panel **(A)** and **(B)**. **(D)** CSP alpha expression in old (24 months) wild type (n = 4) and htau mice (n = 7). CSPalpha expression was normalized against NSE in all panels. Panel **(E)** shows the representative western blots. Means ± s.e.m are shown. ***, p < 0.001.

Together with amyloid plaques, neurofibrillary tangles containing hyperphosphorylated tau aggregates are a pathological hallmark of Alzheimer’s disease. We investigated the levels of CSPalpha in a mouse model of tauopathy that which expresses the entire wild-type human tau gene in the absence of mouse tau [26]. These mutants progressively develop hyperphosphorylated tau and form neurofibrillary tangles predominantly in the cortex and hippocampus. This model also has deficits in basal synaptic transmission, long-term potentiation and memory, and a widespread neuronal loss in old age [27,28]. The tau pathology is visible with biochemical analysis from 3 weeks of age, but neuronal loss is only apparent from 17 months of age onwards. We studied CSPalpha expression in the hippocampus/overlying cortex (Figure 8A, Additional file 4: Figure S3), frontal cortex (Figure 8B) and cerebellum (Figure 8C) of htau mutants and wild-type mice at 3–4 months of age. We found a significant upregulation of CSPalpha expression in hippocampus (Figure 8A, t = 6.539, p < 0.001), frontal cortex (Figure 8B, t = 8.005, p < 0.001) and cerebellum (Figure 8C, t = 5.200, p < 0.001) in 3–4 month-old htau mice relative to controls. At this age there is no neuronal loss in htau mice, and therefore this findings is in agreement with the idea that increased CSPalpha expression may be a neuroprotective mechanism. We also investigated CSPalpha expression at 24 months of age in hippocampal/overlying cortex lysates prepared from htau and wild type mice (Figure 8D). At this age point no differences in the levels of CSPalpha in the hippocampus from wild type and htau mice was apparent (Figure 8D, t = −0.220, p =0.83). Thus, at a time point when neuronal loss is observed, CSPalpha expression is no longer elevated.

## Discussion

The main findings of our study are that 1) expression of CSPalpha is reduced in degenerating forebrain in mild and severe Alzheimer’s disease. This downregulation occurs before synaptophysin levels drop. 2) CSPalpha expression is upregulated in Alzheimer’s disease cerebellum, a brain region protected from synaptic and neuronal loss in Alzheimer’s disease. This upregulation is at a level that occurs in young healthy cerebellum. 3) CSPalpha expression is not upregulated in FTLD cerebellum where neuropathology occurs. 4) In a mouse model of tauopathy CSPalpha upregulation inversely correlates with neurodegeneration. Taken together, these findings provide evidence that CSPalpha is a critical player of synaptic degeneration and synaptic survival in Alzheimer’s disease.

CSPalpha is a p25-regulated protein, and we have previously shown that p25 expression is downregulated in Alzheimer’s disease forebrain [6]. In addition, loss-of-function CSPalpha mutations cause adult-onset Kufs disease that is associated with dementia [11,12]. We therefore speculated that CSPalpha expression could be altered in Alzheimer’s disease. Here we confirm this idea. We found that CSPalpha expression is reduced in hippocampus and STG in severe Alzheimer’s disease. In western blots we detected CSPalpha as two bands due to posttranslational modifications. The posttranslational modifications and the levels of CSPalpha appear variable within a given group. However, when normalized to NSE or synaptophysin and when outliers were excluded (see, Material and methods) significant differences in expression between groups were identified. Our finding that CSPalpha expression is reduced in AD hippocampus and STG is consistent with another study, which was published after we started our project, showing that in Brodmann area 9 of severe Alzheimer’s disease CSPalpha expression is reduced by about 40% [29]. Furthermore, we also detected a downregulation of CSPalpha expression in hippocampus in mild Alzheimer’s disease when CSPalpha amounts were normalized to the synaptic marker synaptophysin. Traditionally, synaptophysin is used as a neuropathological marker of synaptic degeneration in Alzheimer’s disease [3]. However, our finding that CSPalpha levels are reduced without noticeable changes in synaptophysin expression, when relative neuronal expression rather than absolute protein expression is analyzed. When considering the importance of CSPalpha for synaptic function [10], our findings suggest that reduced CSPalpha expression is likely to be involved in the initial stages of synaptic degeneration. Further, for investigating synaptic degeneration in Alzheimer’s disease analysis CSPalpha expression appears more suitable than assessing synaptophysin expression.

CSPalpha, along with its interacting partners Hsc-70 and SGT, is involved in exocytotic mechanisms in presynaptic terminals that are mediated by its interactions with SNARE complexes [9]. Downregulation of CSPalpha may therefore lead to reductions in the number of synaptic vesicles binding at presynaptic membranes, thereby affecting synaptic activity. Further, CSPalpha is also important for endocytosis of synaptic vesicles. CSPalpha interacts with dynamin to facilitate the of dynamin polymerization which is important for endocytotic vesicle fission [17,29]. This is important for normal synaptic function since the number of synaptic vesicles readily available for exocytosis is reduced when there are defects in endocytotic fission [30]. This suggests that defects observed in exocytotic mechanisms in CSPalpha knockout mice could be explained by deficits in CSPalpha-dependent endocytotic mechanisms. Hence, CSPalpha downregulation could lead to loss of function at different stages of synaptic vesicular recycling to contribute to synaptic loss. Additionally, reduced CSPalpha expression is expected to increase BK channel density at synapses, which reduces excitability at presynaptic terminals [18,19]. BK channel activation has been reported to decrease basal synaptic transmission in hippocampal CA1 region of a mouse model of Alzheimer’s disease [31]. Loss of synaptic activity is thought to be lethal for synapses, therefore, the downregulation in CSPalpha expression we observe in Alzheimer’s disease hippocampus could be closely associated with synaptic degeneration and the resulting impaired memory formation in early Alzheimer’s disease.

The second major finding from our study is the identification of CSPalpha upregulation in Alzheimer’s disease cerebellum. The cerebellum is relatively protected from neurodegeneration in Alzheimer’s disease. For example, there is no synaptic and neuronal loss in this area, although there are some diffuse amyloid plaques [1]. The molecular mechanisms that impart neuroprotection to the cerebellum in Alzheimer’s disease are not known. Our results suggest that CSPalpha may be a factor contributing to this neuroprotection. We observed an upregulation of CSPalpha in cerebellum both in mild and severe Alzheimer’s disease. Importantly, we found that the level of upregulation in this region is comparable to the amounts of CSPalpha expression detected in young, healthy cerebellum, in contrast to an age-dependent decrease in CSPalpha expression in normal cerebellum. Additional experimental support for the suggestion that CSPalpha upregulation could be neuroprotective in Alzheimer’s disease comes from our finding that CSPalpha expression is not altered in cerebellum from patients with FTLD, although there is neuropathology in the cerebellum in this disease [25,32,33]. Additional support for our hypothesis comes from analysis of htau mutant mice, where we found that CSPalpha upregulation occurs only at times when no neuronal loss is observed. Taken together, the evidence we present here suggests that CSPalpha upregulation in Alzheimer’s disease cerebellum might be neuroprotective, although in future functional studies in model systems are needed to support this idea.

How could CSPalpha upregulation protect synapses and consequently neurons in Alzheimer’s disease cerebellum? In Alzheimer’s disease forebrain, amyloid-induced aberrations in synaptic activity are one of the causes of synaptic toxicity [34]. In particular, dysfunctional synaptic machinery could be an after-effect of impaired synaptic vesicle trafficking. Aβ oligomers impair synaptic vesicle recycling by hindering endocytosis as well as the formation of fusion-competent vesicles [35]. Furthermore, transgenic mouse studies have suggested that presynaptic degeneration is pivotal in Alzheimer’s disease [36]. Considering the role of CSPalpha in endocytosis and vesicle recycling, an up regulation of CSPalpha in Alzheimer’s disease cerebellum could be a compensatory mechanism that prevents impairments in synaptic vesicle recycling that are induced by factors causing Alzheimer’s disease. This might result in protection of synapses and neurons from degeneration. Functional studies with CSPalpha knockout and upregulation models will provide further insights into the mechanistic basis of our observations.

## Conclusion

Synapse loss in forebrain, but not cerebellum, is a key hallmark of in Alzheimer’s disease. However, the mechanisms causing brain region-dependent synapse loss and protection are unknown. Here we provide evidence that the presynaptic vesicle protein CSPalpha is a critical player in Alzheimer’s disease. In the forebrain CSPalpha expression reduces in the initial stages of synaptic degeneration before synaptophysin levels drop. In cerebellum CSPalpha expression is upregulated both in mild and severe Alzheimer’s disease. This upregulation of CSPalpha is to a level that occurs in young health cerebellum. In a mouse model of tauopathy we confirmed a lack of neuronal loss when CSPalpha expression is elevated. Taken together, these findings point to critical role for CSPalpha in synaptic degeneration and protection in Alzheimer’s disease.

## Material and methods

### Post-mortem human brain samples

Brain tissues in 10% (v/v) formalin-fixed, paraffin-embedded tissue blocks and as frozen tissues were available from the Medical Research Council (MRC) London Neurodegenerative Diseases Brain Bank (Institute of Psychiatry, King’s College London, UK). All tissue collection and processing was carried out under the regulations and licensing of the Human Tissue Authority and in accordance with the Human Tissue Act, 2004. Frozen samples were received in two sets for western blot analysis. The first set contained hippocampal tissue from control subjects, mild Alzheimer’s disease (Braak stages 1–2) and severe Alzheimer’s disease (Braak stages 5–6) [n = 7 for each group], as well as superior temporal gyrus (STG) samples from controls and severe Alzheimer’s disease [n = 7 and n = 9, respectively]. The second set comprised hippocampus, STG and cerebellum samples from control, mild and severe Alzheimer’s disease patients (n = 5 for each group). To increase the sample size of cerebellum, a new cohort (n = 5 per group) was later added to the analysis. Cerebellum samples (n = 5) were obtained from frontotemporal lobar degeneration (FTLD) patients. Cerebellum tissues were also obtained from healthy subjects less than 30 years old (n = 6) and older than 90 years (n = 7). Additional file 5: Table S1 summarizes the details.

### Lysate preparation from human brain samples

Frozen brain samples were lysed at 4°C in RIPA lysis buffer (Santa Cruz Biotechnology, Inc., USA) consisting of 0.1% SDS, 1% Nonidet P-40, 0.5% sodium deoxycholate and 0.004% sodium azide in TBS (pH 7.5). Protease inhibitors cocktail, sodium orthovanadate and α-toluenesulphonyl fluoride in DMSO (all Santa Cruz Biotechnology, Inc., USA) were added to the buffer, diluted to 0.01%. The SDS concentration was increased to 0.25%. Approximately 100 mg of brain tissue was lysed in 300 μl buffer. Samples were homogenized using a dounce homogenizer (12 up and down strokes, 700 rotations per minute) at 4°C, and centrifuged at 3,000 rpm for 10 minutes at 4°C. Supernatants were immunoblotted and the bands from protein of interest were normalized with housekeeping proteins.

### Mouse brain samples

Frontal cortex, cerebellum and hippocampus/overlying cortex was isolated from 3–4 month old human tau (htau) mice in the C57BL/6 J background (Jackson Laboratories, Bar Harbor, Maine USA; Stock number: 005 491). Mice were genotyped by PCR to confirm the presence of the human *MAPT* transgene and the mouse *Mapt null* background using primers for the *MAPT* gene (forward 5′-ACTTTGAACCAGGATGGCTGAGCCC-3′, reverse 5′-CTGTGCATGGCTGTCCCTACCTT-3′), and the mouse *Mapt* gene (forward 5′-CTCAGCATCCCACCTGTAAC-3′, reverse 5′-CCAGTTGTGTATGTCCACCC-3′), as described in [20]. The primers for the disrupted *Mapt* gene were: forward 5′-AAGTTCATCTGCACCACCG-3′, reverse 5′-TCCTTGAAGAAGATGGTG CG-3′.

Mice were housed on 12 h light:12 h dark cycles with food and water available *ad libitum*. Mice were killed by cervical dislocation and brain regions snap frozen on dry ice. All animal procedures were conducted in accordance with the UK Home Office, Animals Scientific Procedures Act 1986.

### Lysate preparation from mouse brain samples

Frozen tissue was homogenised at 100 mg/ml in 2× sample buffer (0.5 M Tris–HCl, pH 6.8, 4.4% SDS, 20% glycerol, 2% 2-mercaptoethanol, 0.01% bromophenol blue, and complete mini-protease inhibitor cocktail (Roche Products Ltd., UK). Following brief sonication, homogenates were centrifuged at 25,000 g for 20 minutes at 4°C, and the supernatant was collected.

### Western blot analysis

The same protein amounts were separated on criterion TGX precast gels (4-15%) gels (BioRad) and the protein was transferred onto a methanol activated PVDF membrane (BioRad), using standard protocols. Non-specific binding was blocked by 5% non-fat dried milk in TBST for 1 hour at room temperature. Subsequently, membranes were incubated overnight at 4°C in primary antibody solution prepared in blocking buffer. After three ten minute washes in TBST at room temperature, membranes were incubated for two hours at room temperature with horse-radish peroxidase conjugated secondary antibodies in blocking buffer. After three ten minute washes with TBST, the membrane was incubated for 3 minutes in ECL reagent (Thermo Scientific) and then exposed to an X-ray film (Amersham) in the linear range. To probe the membranes with other primary antibodies, membranes were treated with a stripping buffer (Santa Cruz Biotechnology) for one hour at room temperature, followed by three washes with TBST of 10 minutes each and subsequent labelling as described above. Primary antibodies against CSPalpha (1:50,000, AB1576 Merck Millipore), synaptophysin (1:1000, 4329 Cell Signalling Technology) and neuron specific enolase (NSE) (1:60,000, AB 951 Merck Millipore) were used. Signals were analyzed using ImageJ software (NIH). With the antibodies against CSPalpha sometimes two bands were detected in human post-mortem brain. These bands are not CSPbeta and CSPgamma since these proteins are not expressed in brain [10]. For standardization CSPalpha was normalized against NSE or synaptophysin.

### Immunohistochemistry

Sections of human brain of 7 μm thickness were cut from paraffin-embedded tissue blocks. Sections were deparaffinised in xylene and rehydrated in ethanol. Endogenous peroxidase activity was blocked by incubation of sections with 2.5% H_2_O_2_ in methanol. To enhance antigen retrieval sections were exposed to citrate buffer (2.94 g/L, pH 6.0) for 16 minutes microwave treatment (6 minutes high, two 5 minutes simmer). After blocking in normal swine serum (DAKO Ltd), primary antibodies against CSPalpha (1:500, AB1576 Merck Millipore), and synaptophysin (1:100, SY38 DAKO Ltd) were applied overnight at 4°C. Following rinsing and two five minutes washes in TBS, sections were incubated with appropriate biotinylated secondary antibodies (1:100, Swine anti-rabbit immunoglobulin/biotinylated, E0353 DAKO Ltd), followed by incubation with avidin:biotin enzyme complex (Vectastain Elite ABC kit, Vector Laboratories, Peterborough, UK). Following washing, sections were incubated for 10–15 min with 0.5 mg/ml 3,3′-diaminobenzidine chromogen (Sigma-Aldrich Company Ltd, Dorset UK) in Tris-buffered saline (pH 7.6) containing 0.05% H_2_O_2_. Sections were counterstained with Harris’s haematoxylin.

### Statistical analysis

Un-paired t tests were used for comparison of data from samples tested in one western blot. – In some cases, the sample size was so large so that data from more than one western blot needed to be pooled. To allow pooling of data by linear regression, the following equation was used -

$$ {\left(\frac{\mathrm{CSP}}{\begin{array}{l}\mathrm{Normalization}\;\mathrm{Marker}\\ {}\left(\mathrm{N}\mathrm{S}\mathrm{E},\mathrm{Synaptophysin}\right)\end{array}}\right)}_{\mathrm{i}}={\upbeta}_0+{\upbeta}_1\times {\mathrm{X}}_{1\mathrm{i}}+{\upbeta}_2\times {\mathrm{X}}_{2\mathrm{i}}+{\upvarepsilon}_{\mathrm{i}} $$

Where X_1i_ is the categorical predictor coding for the group difference (e.g. Control versus Severe), and X_2i_ is the categorical predictor coding for the different experiments (“*1*^*st*^*cohort*” versus “*2*^*nd*^*cohort*”).

This regression model allowed us to pool the CSPalpha score from two different set of samples by eliminating the contribution made by the difference in experimental conditions. This analysis was performed using SPSS (version 20), which provides the output as an ANOVA score. The contribution and the significance of the factor of interest (e.g. the disease pathology) to the overall significance is subsequently determined by the score from this output. The level of significance for the analysis was 0.05 and outliers were decided by using mean ± 4*SD as threshold. See Additional file 6 in supplementary information for analysis of post-mortem brain tissue.

## Additional files

Additional file 1: Figure S1. Synaptophysin protein expression in reference to NSE expression is unchanged in Alzheimer’s disease hippocampus. **(A)** Synaptophysin expression in post-mortem hippocampus from patients with severe AD (n = 12) and control subjects (n = 12) was normalized against the neuron specific house keeping marker protein NSE. **(B)** Synaptophysin expression in post-mortem hippocampus from patients with mild AD (n = 12) and control subjects (n = 12) was normalized against NSE. Means ± s.e.m. are shown. *, p < 0.05; **, p < 0.01. Additional file 2: Figure S4. Decreased CSPalpha immunostaining in Alzheimer’s disease hippocampus and STG. Fixed hippocampal sections from a patient with severe Alzheimer’s disease **(D)** and an age-matched control subject **(C)** were probed with anti-CSPalpha antibodies for analysis of CSPalpha expression.Sections from the hippocampus with the granule cell layer show decreased immunoreactivity with a synaptic staining pattern in Alzheimer’s disease as compared to the control case. A similar analysis was carried out for expression in superior temporal gyrus (STG) from a patient with severe Alzheimer’s disease **(B)** and an age-matched control subject **(A)**. In the STG there is decreased immunoreactivity in Alzheimer’s disease as compared to control. Haematoxylin counterstain is included in the analysis. Original magnification: ×400. Scale bars represent 20 μm. Additional file 3: Figure S2. CSPalpha immunostaining in cerebellum is typical for synaptic expression. Immunohistochemical sections of cerebellar dentate nucleus region from a control patient were immunostained with anti-CSPalpha antibodies **(A)** and with antibodies against the synaptic marker synaptophysin **(B)**. This comparison indicated that the CSPalphaimmunostaining is synaptic as obtained for synaptophysin immunostaining. Scale bars represent 200 μm. Additional file 4: Figure S3. Synaptophysin levels relative to NSE expression are unchanged in hTau mutant mouse hippocampus. Synaptophysin expression in hippocampus of young (3–4 months) wild type (n = 6) and hTau mice (n = 6) was normalized against NSE in all panels. Means ± s.e.m are shown. Additional file 5: Table S1. Details of post-mortem brain tissues for western blots. PMD refers to post-mortem delay. Additional file 6:
**Statistical analysis of the effect of age, gender and post mortem delay on post-mortem brain tissues used in this study.**

## Abbreviations

CSP: Cysteine String Protein
STG: Superior Temporal Gyrus
FTLD: Fronto Temporal Lobar Degeneration
NSE: Neuronal Specific Enolase
