# Supplementary figures and images for: Evidence that the presynaptic vesicle protein CSPalpha is a key player in synaptic degeneration and protection in Alzheimer’s disease
Source: Mol Brain. 2015 Jan 29;8:6. doi: 10.1186/s13041-015-0096-z (PMC4314762; doi:10.1186/s13041-015-0096-z)

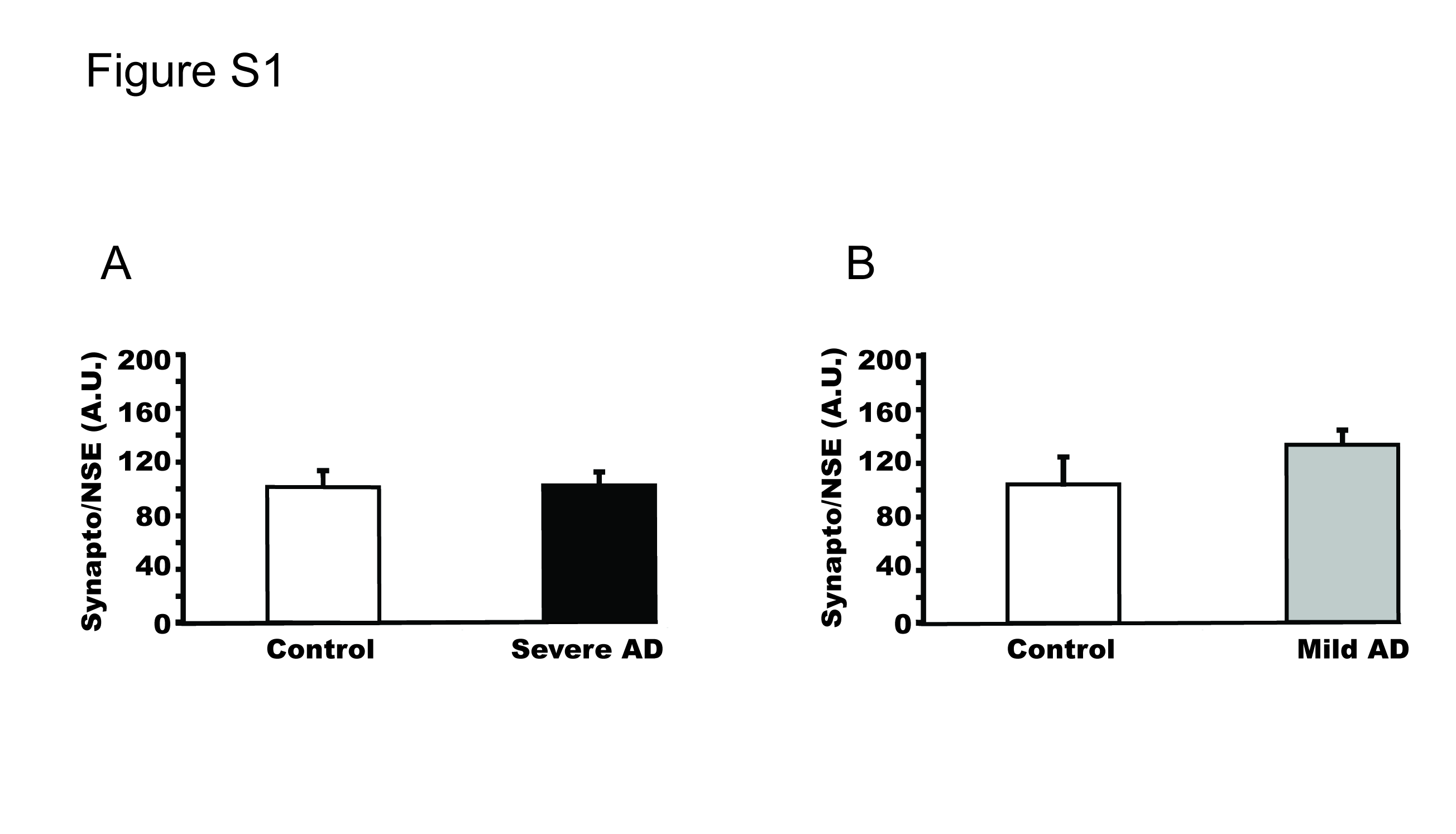

Supplement: Additional file 1: Figure S1. — Synaptophysin protein expression in reference to NSE expression is unchanged in Alzheimer’s disease hippocampus. (A) Synaptophysin expression in post-mortem hippocampus from patients with severe AD (n = 12) and control subjects (n = 12) was normalized against the neuron specific house keeping marker protein NSE. (B) Synaptophysin expression in post-mortem hippocampus from patients with mild AD (n = 12) and control subjects (n = 12) was normalized against NSE. Means ± s.e.m. are shown. *, p < 0.05; **, p < 0.01. [file 13041_2015_96_MOESM1_ESM.tiff]

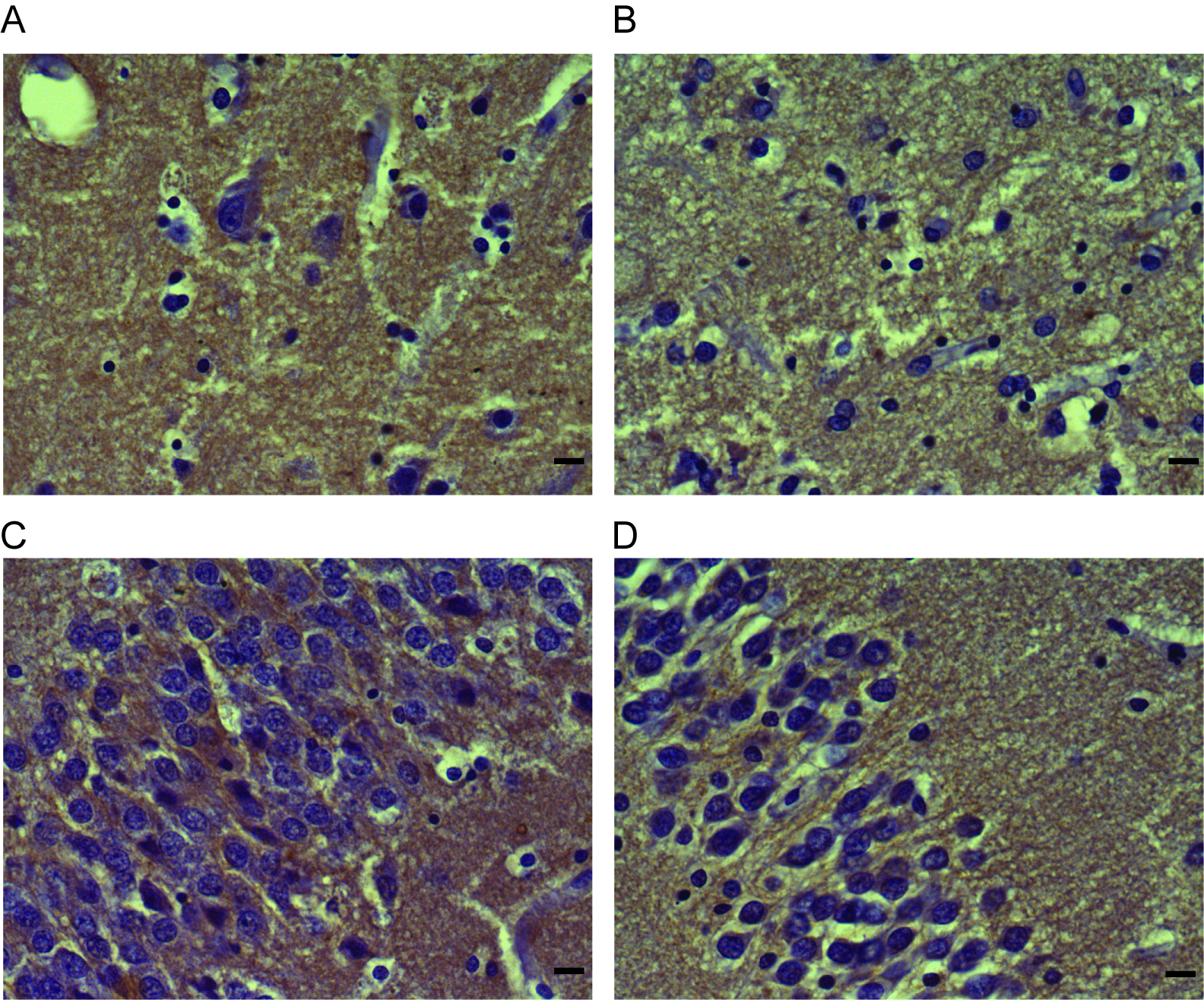

Supplement: Additional file 2: Figure S4. — Decreased CSPalpha immunostaining in Alzheimer’s disease hippocampus and STG. Fixed hippocampal sections from a patient with severe Alzheimer’s disease (D) and an age-matched control subject (C) were probed with anti-CSPalpha antibodies for analysis of CSPalpha expression.Sections from the hippocampus with the granule cell layer show decreased immunoreactivity with a synaptic staining pattern in Alzheimer’s disease as compared to the control case. A similar analysis was carried out for expression in superior temporal gyrus (STG) from a patient with severe Alzheimer’s disease (B) and an age-matched control subject (A). In the STG there is decreased immunoreactivity in Alzheimer’s disease as compared to control. Haematoxylin counterstain is included in the analysis. Original magnification: ×400. Scale bars represent 20 μm. [file 13041_2015_96_MOESM2_ESM.tiff]

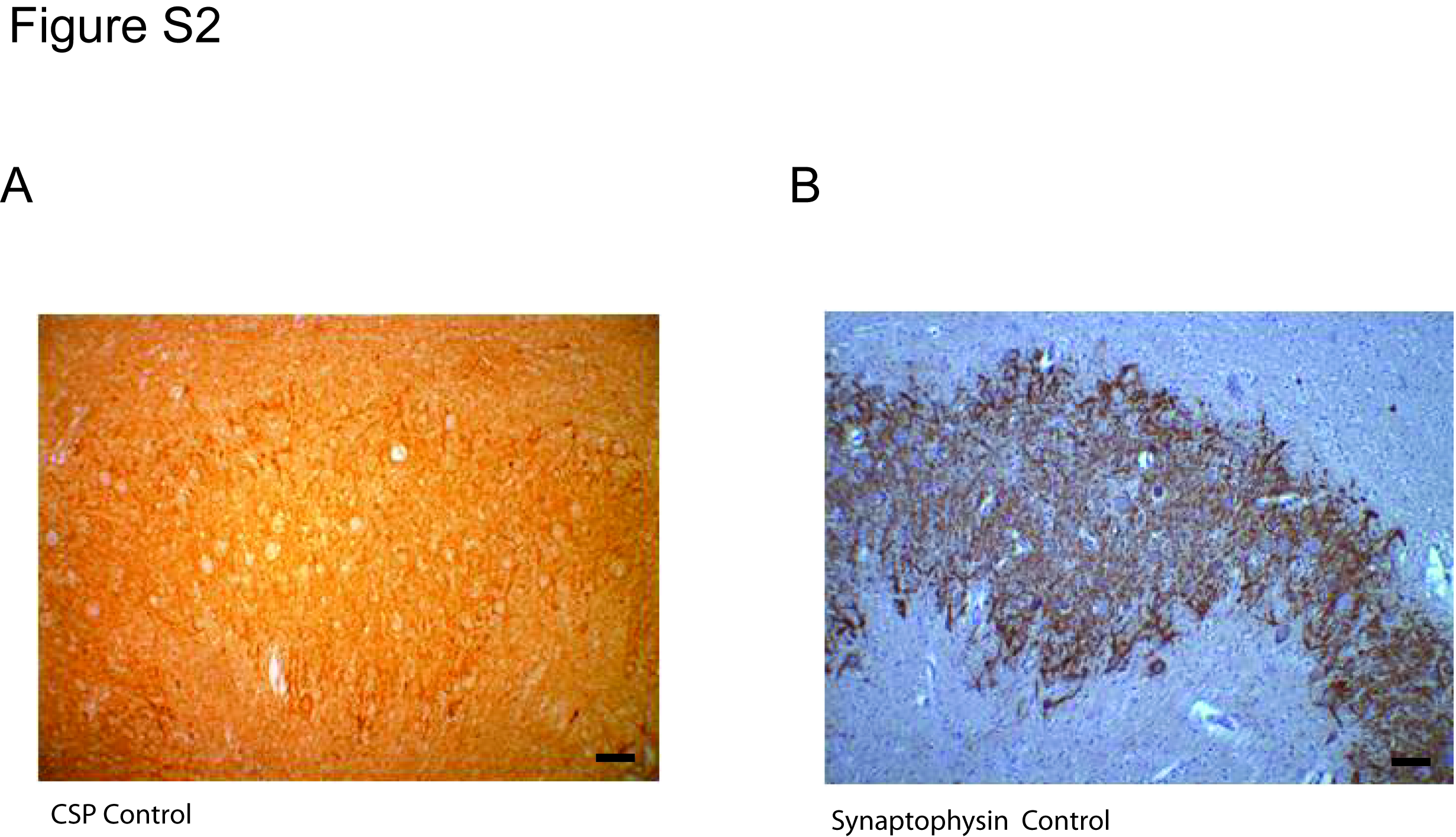

Supplement: Additional file 3: Figure S2. — CSPalpha immunostaining in cerebellum is typical for synaptic expression. Immunohistochemical sections of cerebellar dentate nucleus region from a control patient were immunostained with anti-CSPalpha antibodies (A) and with antibodies against the synaptic marker synaptophysin (B). This comparison indicated that the CSPalphaimmunostaining is synaptic as obtained for synaptophysin immunostaining. Scale bars represent 200 μm. [file 13041_2015_96_MOESM3_ESM.tiff]

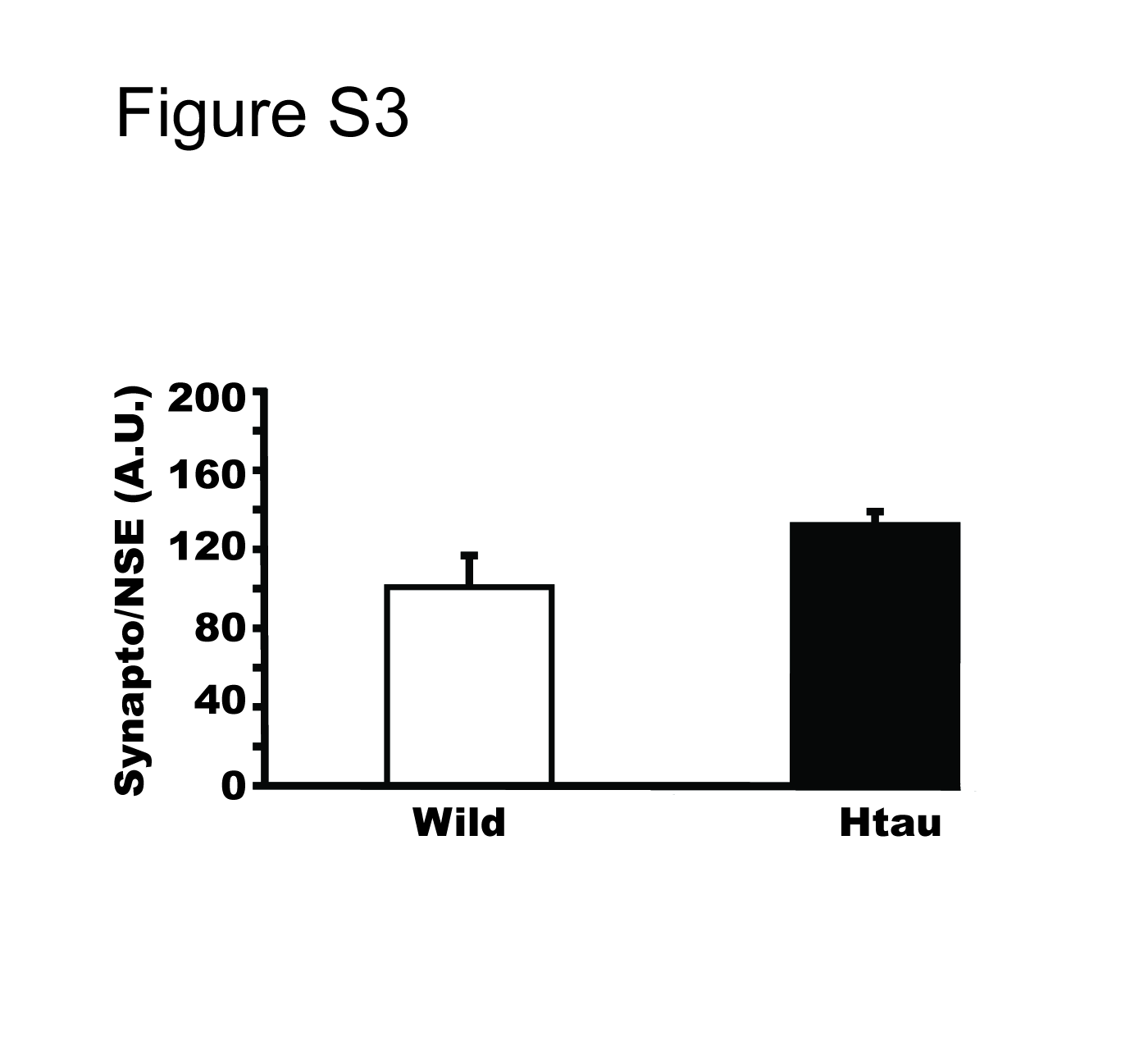

Supplement: Additional file 4: Figure S3. — Synaptophysin levels relative to NSE expression are unchanged in hTau mutant mouse hippocampus. Synaptophysin expression in hippocampus of young (3–4 months) wild type (n = 6) and hTau mice (n = 6) was normalized against NSE in all panels. Means ± s.e.m are shown. [file 13041_2015_96_MOESM4_ESM.tiff]
