# Supplementary material for: Evidence that the presynaptic vesicle protein CSPalpha is a key player in synaptic degeneration and protection in Alzheimer’s disease
Source: Mol Brain. 2015 Jan 29;8:6. doi: 10.1186/s13041-015-0096-z (PMC4314762; doi:10.1186/s13041-015-0096-z)
Supplement: Additional file 5: Table S1. — Details of post-mortem brain tissues for western blots. PMD refers to post-mortem delay. [file 13041_2015_96_MOESM5_ESM.doc]

**Table S1. Details of post-mortem brain tissues for western blots.** PMD refers to post-mortem delay.

**Hippocampus** -

| S.No | Pathological state | Sex | Age(Years) | PMD( Hours) |
| --- | --- | --- | --- | --- |
| 1 | Control | M | 81 | 18 |
| 2 | Control | F | 92 | 17 |
| 3 | Control | M | 78 | 10 |
| 4 | Control | M | 85 | 16 |
| 5 | Control | F | 76 | 28 |
| 6 | Control | M | 65 | 24 |
| 7 | Control | M | 86 | 6 |
| 8 | Control | F | 72 | 24 |
| 9 | Control | F | 55 | 24 |
| 10 | Control | F | 80 | 31 |
| 11 | Control | F | 71 | 30 |
| 12 | Control | M | 77 | 29 |
| 13 | Mild AD | M | 81 | 12 |
| 14 | Mild AD | F | 92 | 9 |
| 15 | Mild AD | F | 80 | 3 |
| 16 | Mild AD | F | 55 | 12 |
| 17 | Mild AD | F | 81 | 17 |
| 18 | Mild AD | F | 81 | 16.5 |
| 19 | Mild AD | F | 82 | 13 |
| 20 | Mild AD | M | 64 | 16 |
| 21 | Mild AD | F | 83 | 24 |
| 22 | Mild AD | M | 81 | 3 |
| 23 | Mild AD | M | 90 | 5.5 |
| 24 | Mild AD | F | 94 | 21 |
| 25 | Severe AD | M | 64 | 23 |
| 26 | Severe AD | F | 68 | 11 |
| 27 | Severe AD | M | 80 | 15 |
| 28 | Severe AD | F | 69 | 16 |
| 29 | Severe AD | M | 77 | 10 |
| 30 | Severe AD | F | 69 | 16.3 |
| 31 | Severe AD | F | 79 | 24 |
| 32 | Severe AD | F | 71 | 21 |
| 33 | Severe AD | F | 82 | 4.5 |
| 34 | Severe AD | F | 80 | 4.3 |
| 35 | Severe AD | F | 88 | 19 |
| 36 | Severe AD | M | 75 | 17 |

**Superior Temporal Gyrus** -

| S.No | Pathological state | Sex | Age(Years) | PMD( Hours) |
| --- | --- | --- | --- | --- |
| 1 | Control | F | 55 | 24 |
| 2 | Control | M | 55 | 24 |
| 3 | Control | M | 65 | 24 |
| 4 | Control | M | 69 | 24 |
| 5 | Control | M | 86 | 6 |
| 6 | Control | M | 65 | 24 |
| 7 | Control | M | 71 | 5 |
| 8 | Control | M | 81 | 18 |
| 9 | Control | F | 92 | 17 |
| 10 | Control | M | 78 | 10 |
| 11 | Control | M | 85 | 16 |
| 12 | Control | F | 76 | 28 |
| 13 | Severe AD | F | 69 | 16.3 |
| 14 | Severe AD | F | 71 | 21 |
| 15 | Severe AD | F | 80 | 4 |
| 16 | Severe AD | F | 81 | 24 |
| 17 | Severe AD | F | 82 | 4.5 |
| 18 | Severe AD | F | 82 | 12 |
| 19 | Severe AD | F | 88 | 19 |
| 20 | Severe AD | F | 91 | 23 |
| 21 | Severe AD | M | 75 | 17 |
| 22 | Severe AD | M | 64 | 23 |
| 23 | Severe AD | F | 68 | 11 |
| 24 | Severe AD | M | 80 | 15 |
| 25 | Severe AD | F | 69 | 16 |
| 26 | Severe AD | M | 77 | 10 |

**Cerebellum** -

| S.No | Pathological state | Sex | Age(Years) | PMD( Hours) |
| --- | --- | --- | --- | --- |
| 1 | Control | M | 73 | 23 |
| 2 | Control | F | 92 | 23 |
| 3 | Control | F | 55 | 12 |
| 4 | Control | M | 77 | 11 |
| 5 | Control | M | 54 | 30 |
| 6 | Control | M | 81 | 18 |
| 7 | Control | F | 92 | 17 |
| 8 | Control | M | 78 | 10 |
| 9 | Control | M | 85 | 16 |
| 10 | Control | F | 76 | 28 |
| 11 | Mild AD | M | 81 | 12 |
| 12 | Mild AD | F | 92 | 9 |
| 13 | Mild AD | F | 80 | 3 |
| 14 | Mild AD | F | 55 | 12 |
| 15 | Mild AD | F | 81 | 17 |
| 16 | Mild AD | M | 93 | 14 |
| 17 | Mild AD | F | 84 | 24 |
| 18 | Mild AD | F | 92 | 20 |
| 19 | Mild AD | M | 92 | 11 |
| 20 | Mild AD | M | 63 | 16 |
| 21 | Severe AD | M | 70 | 20 |
| 22 | Severe AD | F | 71 | 18 |
| 23 | Severe AD | F | 92 | 11 |
| 24 | Severe AD | F | 61 | 3 |
| 25 | Severe AD | F | 95 | 13 |
| 26 | Severe AD | M | 64 | 23 |
| 27 | Severe AD | F | 68 | 11 |
| 28 | Severe AD | M | 80 | 15 |
| 29 | Severe AD | F | 69 | 16 |
| 30 | Severe AD | M | 77 | 10 |

**Cerebellum (FTLD)** -

| S.No | Pathological state | Sex | Age(Years) | PMD( Hours) |
| --- | --- | --- | --- | --- |
| 1 | Control | M | 73 | 23 |
| 2 | Control | F | 92 | 23 |
| 3 | Control | F | 55 | 12 |
| 4 | Control | M | 77 | 11 |
| 5 | Control | M | 54 | 30 |
| 6 | FTD | F | 85 | 24 |
| 7 | FTD | M | 69 | 6 |
| 8 | FTD | M | 71 | 14 |
| 9 | FTD | F | 70 | 16 |
| 10 | FTD | M | 70 | 7 |

**Cerebellum (healthy ageing)** -

| S.No | Age Category | Sex | Age(Years) | PMD( Hours) |
| --- | --- | --- | --- | --- |
| 1 | Young Control | M | 18 | 24.5 |
| 2 | Young Control | M | 22 | 45 |
| 3 | Young Control | M | 21 | 37 |
| 4 | Young Control | M | 16 | 14 |
| 5 | Young Control | M | 25 | 18 |
| 6 | Young Control | F | 26 | 10 |
| 7 | Old Control | F | 102 | 44 |
| 8 | Old Control | M | 97 | 44 |
| 9 | Old Control | F | 99 | 32 |
| 10 | Old Control | F | 96 | 16 |
| 11 | Old Control | F | 92 | 9 |
| 12 | Old Control | F | 92 | 23 |
| 13 | Old Control | M | 95 | 44 |
