# Supplementary material for: Evidence that the presynaptic vesicle protein CSPalpha is a key player in synaptic degeneration and protection in Alzheimer’s disease
Source: Mol Brain. 2015 Jan 29;8:6. doi: 10.1186/s13041-015-0096-z (PMC4314762; doi:10.1186/s13041-015-0096-z)
Supplement: Additional file 6: — Statistical analysis of the effect of age, gender and post mortem delay on post-mortem brain tissues used in this study. [file 13041_2015_96_MOESM6_ESM.doc]

**Supplementary information**

**Supplementary data 1**. **Statistical analysis of the effect of age, gender and post mortem delay on post-mortem brain tissues used in this study** –

Since the data used in this project involved pooling the data from two different sets, and hence utilized the regression analysis model for statistical studies, it was not suitable to perform a correlation study between different aforesaid parameters and the protein levels observed in post-mortem tissues. Hence, we adopted the strategy of testing for a difference between gender, age and post-mortem delay (PMD) of patient samples grouped into different pathological state of disease– control, mild, severe Alzheimer’s disease.

***Hippocampus* Alzheimer’s disease *samples –***

A) Effect of gender – Pearson’s chi-square test was performed to discover the relationship between the categorical variable (Gender and Pathological state). The p=0.627, shows there was no statistically significant association between gender and pathological state.

B) Effect of age – One way ANOVA showed no difference in age between control, mild and severe groups (F(2,33) = 0.961 , p=0.393).

C) Effect of PMD – One-way ANOVA showed an association between post-mortem delay and the pathological state of patients (F (2,33) = 4.812, p=0.015). Tukey’s posthoc test revealed that the post-mortem delay was not significantly different between the control and severe pathological state group (p=0.091). It was also not different between the mild and severe pathological state group (p=0.686). However, the post-mortem delay between control and mild pathological state showed significant difference (p= 0.014). The post-mortem delay in control (21.4 ± 2.3 hours) was significantly longer than in mild Alzheimer’s disease patients hippocampus (12.7 ± 1.9 hours). It is unlikely that this difference may have impacted on our result as CSPalpha expression was a significantly increased in control hippocampus group as compared to mild Alzheimer’s disease hippocampus.

***STG Alzheimer’s disease samples –***

A) Effect of gender - Pearson’s chi-square test was performed to discover the relationship between the categorical variable (Gender and pathological state). The p=0.018, showed a statistically significant association between gender and its pathological state. Since none of the studied proteins have been reported to have sex linkages, the possibility of gender differences having an impact on our results appears negligible.

B) Effect of age – Independent sample two tailed t-test showed there was no significant difference in the age between the control and severe STG groups (t= -0.959, p=0.347)

C) Effect of PMD – Independent sample two tailed t-test showed there was no significant difference in the postmortem delay between the control and STG groups (t= 1.046, p=0.306)

***Cerebellum Alzheimer’s disease samples –***

A) Effect of gender - Pearson’s chi-square test was performed to discover the relationship between the categorical variable (gender and pathological state). The p=0.585, showed no statistically significant association between the individuals gender and its pathological state.

B) Effect of age – One way ANOVA showed there was no significant difference in the age between the control, mild and severe AD cerebellum (F (2,27) = 0.756, p=0.479).

C) Effect of PMD – One-way ANOVA showed that there was no significant difference in the post-mortem delay between the control, mild and severe AD cerebellum (F(2,27)=2.066, p=0.146).

***Cerebellum FTLD samples* –**

A) Effect of gender - Pearson’s chi-square test was performed to discover the relationship between the categorical variable (Gender and pathological state). The p=1.0, showed no statistically significant association between the individuals gender and its pathological state.

B) Effect of age – Independent sample two tailed T-test showed there was no significant difference in the age between the control and FTLD cerebellum, (t = - 0.361, p=0.728).

C) Effect of PMD – Independent sample two tailed t-test showed that there was no significant difference in the post-mortem delay between the control and FTLD cerebellum. (t=1.309, p=0.227).

***Cerebellum ageing study samples* –**

Since age was different in the two groups within this study (young and old cerebellum), the effect of gender and post-mortem delay was ascertained.

A) Effect of gender - Pearson’s chi-square test was performed to discover the relationship between the categorical variable (gender and pathological state). The p=0.048, shows a statistically significant association between the individuals gender and its pathological state. Since CSPα does not shows sex-linked inheritance, it is possible that this slight significance is due to the small sample size and this significance might disappear with increase in number of cerebellar samples.

B) Effect of post-mortem delay - Independent sample two tailed T-test showed that there was no significant difference in the post-mortem delay between the young control and old control cerebellum (t=-0.701, p=0.498).
